# Pax2 coordinates epithelial morphogenesis and cell fate in the inner ear

**DOI:** 10.1016/j.ydbio.2010.07.007

**Published:** 2010-09-15

**Authors:** Nicolas A.D. Christophorou, Michael Mende, Laura Lleras-Forero, Timothy Grocott, Andrea Streit

**Affiliations:** Department of Craniofacial Development, King's College London, Guy's Campus, Tower Wing Floor 27, London SE1 9RT, UK

**Keywords:** Cell adhesion, Cell shape, Chick, Eya1, Gata3, Invagination, Placode, Transcription factor

## Abstract

Crucial components of the vertebrate eye, ear and nose develop from discrete patches of surface epithelium, called placodes, which fold into spheroids and undergo complex morphogenesis. Little is known about how the changes in cell and tissue shapes are coordinated with the acquisition of cell fates. Here we explore whether these processes are regulated by common transcriptional mechanisms in the developing ear. After specification, inner ear precursors elongate to form the placode, which invaginates and is transformed into the complex structure of the adult ear. We show that the transcription factor Pax2 plays a key role in coordinating otic fate and placode morphogenesis, but appears to regulate each process independently. In the absence of Pax2, otic progenitors not only lose otic marker expression, but also fail to elongate due to the loss of apically localised N-cadherin and N-CAM. In the absence of either N-cadherin or N-CAM otic cells lose apical cell–cell contact and their epithelial shape. While misexpression of Pax2 leads to ectopic activation of both adhesion molecules, it is not sufficient to confer otic identity. These observations suggest that Pax2 controls cell shape independently from cell identity and thus acts as coordinator for these processes.

## Introduction

The vertebrate inner ear develops from a simple epithelium, the otic placode, next to the hindbrain (Ohyama et al., 2007; Riley and Phillips, 2003; Whitfield et al., 2002). The placode invaginates to form the otic vesicle, which folds into the complex structure of the mature ear over time. Otic fate is induced by signals from adjacent tissues: members of the fibroblast growth factor (FGF) family induce the pre-otic field, which is then further refined through a combination of Notch and Wnt signalling (Freter et al., 2008; Jayasena et al., 2008; Ladher et al., 2000; Leger and Brand, 2002; Maroon et al., 2002; Ohyama et al., 2006; Phillips et al., 2001; Wright and Mansour, 2003). In response to these signals, cells begin to express a series of transcription factors that successively impart otic identity: among the earliest factors are *Foxi1* and *Dlx* genes, followed *Pax8*, *Pax2* and *Sox3* in the otic-epibranchial territory and *Eya1*, *Gata3*, *Gbx2* and *Sox9* in the otic region (for review see: Ohyama et al., 2007; Riley and Phillips, 2003; Schlosser, 2006; Ladher et al., 2010). Studies in zebrafish have led to a model where Foxi1 acts upstream of *Pax8*, while Dlx proteins activate *Pax2* slightly later and both Pax proteins then cooperate to promote otic fate (Hans et al., 2004; Mackereth et al., 2005; Nissen et al., 2003; Solomon et al., 2003, 2004), but most of these interactions remain to be verified in amniotes. For example, while Pax2 mutant mice show late ear defects, the ears of Pax8 mutants develop relatively normally and double mutant phenotypes have not been examined (Burton et al., 2004; Christ et al., 2004; Torres et al., 1996). Thus, in amniotes, a role for Pax genes in otic specification has not been established.

Following otic induction ectodermal cells undergo morphological changes critical for the formation of the placode proper and for its subsequent development into a functional ear. Like the neural plate (Colas and Schoenwolf, 2001; Schoenwolf and Franks, 1984; Wallingford, 2005), placode cells first lengthen along their apical–basal axis to form a columnar epithelium and invagination is initiated by apical constriction leading to vesicle formation (Bancroft and Bellairs, 1977; Hilfer et al., 1989; Schook, 1980a,b). This process is driven by contraction of the apically localised F-actin network by myosin (Sai and Ladher, 2008; for review: Sawyer et al., 2010). The subsequent morphogenetic processes that transform the otic vesicle into the intricate structure of the mature ear are poorly understood, although differential proliferation and apoptosis have been implicated (Lang et al., 2000). These morphogenetic events must be tightly coordinated with cell fate acquisition to form a functional ear and again, little is known about the molecular mechanisms responsible.

Interestingly, Pax2 is prominently expressed in the ear and elsewhere at sites where tissue outgrowth and shaping takes place (Dressler et al., 1990; Grote et al., 2006; Nornes et al., 1990; Rajakumar and Chamberlin, 2007), raising the possibility that Pax2 plays a role in regulating these events. Here we test the hypothesis that Pax2 coordinates otic identity and morphogenesis. We find that it is required for the expression of early otic markers and that it independently controls epithelial integrity of the placode. Downstream of Pax2, N-cadherin and N-CAM are required to maintain apical cell adhesion between otic cells as a prerequisite for placode invagination.

## Materials and methods

### Embryo culture and electroporation

Fertile hens' eggs were incubated in a humidified incubator at 38 °C until they had reached the appropriate stage (Hamburger and Hamilton, 1951) (HH). Expression vectors and morpholinos were transfected into the head ectoderm using electroporation (McLarren et al., 2003; Mende et al., 2008) and maintained in New culture (New, 1955; Stern and Ireland, 1981) for 16–27 h. Embryos were fixed in 4% paraformaldehyde, 2 mM EGTA in phosphate buffered saline (PBS) overnight at 4 °C for in situ hybridisation or in 4% paraformaldehyde in PBS for 30 min at room temperature for immunocytochemistry.

### Morpholinos and expression constructs

All morpholinos (MO) were labelled with fluorescein conjugates (Gene Tools). MOs targeting Pax2 (Mende et al., 2008) and N-cadherin (Shiau and Bronner-Fraser, 2009) were described previously. For N-CAM two different MOs were designed targeting the translation start site (MO1: 5′-GCCGCTCCGAAATAGCCGTTCCGTG-3′) and a splice blocking MO targeting the boundary of exon 7 and intron 7 (MO2: 5′-AACAGGCAAAAGCTCACCAAAGACT-3′). Each N-CAM MO was tested separately for efficient knock down (n = 7 for MO1, n = 8 MO2); the experiments shown in the results section used electroporation of both MOs simultaneously. As control we used sense MO or standard control MO (Gene Tools). The coding sequences of mouse Pax2 (Dressler et al., 1990) and chick Sox2 (Rex et al., 1997) were cloned into pCAB-IRES-GFP (McLarren et al., 2003) to generate Pax2 and Sox2 expression constructs. We confirmed that the Sox2 expression construct produces Sox2 protein by performing Sox2 antibody staining (R&D) after electroporation into the cranial ectoderm.

### In situ hybridisation and immunocytochemistry

Whole mount in situ hybridisation was performed as previously described (Streit et al., 1998) using DIG-labelled anti-sense probes for Eya1 (Chest668D18), Gata3 (Sheng and Stern, 1999), Pax2 (Streit, 2002), Sox2 and -3 (Rex et al., 1997). Antibody staining was performed on cryosections as previously described (Bailey et al., 2006) using polyclonal antibodies against Pax2 (Zymed) and β-catenin (Abcam) and monoclonal antibodies against α-catenin (BD Biosciences), N-cadherin (Sigma) and N-CAM (5e; Developmental Hybridoma Bank). Appropriate secondary antibodies were coupled to Alexa 488, Alexa 594 or Cy5 (Invitrogen). Alexa 488 phalloidin was used to label actin and nuclei were visualised using DAPI. Sections were analysed using a Leica TCS SP5 confocal microscope. The elongation index (length/width ratio) was determined in individual GFP or MO^+^ otic placode cells by measuring their maximum length and width. Mean values and standard deviation were determined and the Mann–Whitney Rank Sum test was used to determine statistical significance.

### Identification of putative Pax2 binding sites on the otic N-cadherin enhancer

Genomic sequences for human, mouse and chick N-cadherin loci were downloaded from the Ensembl genome browser. A VistaPlot alignment of the N-cadherin loci was cross-referenced to the position and sequence of the known chick En2-DP enhancer (Matsumata et al., 2005). VistaPlot yielded a ClustalW alignment for a conserved non-coding sequence corresponding to the En2-DP enhancer, from which a consensus enhancer sequence was derived. Putative conserved transcription factor binding sites were identified within the consensus enhancer sequence according to the consensus recognition sequences for Gata [A/T]GATA[A/G], Pax2 TNGTCA[C/T]GC[A/G]TGA and SoxB1, ATTGTG. Of the putative binding sites identified, only those with the highest cross-species conservation were annotated, as follows: Gata > 80% identity, Pax2 > 50% identity and SoxB1 > 65% identity. These identity thresholds were chosen to reflect the length and heterogeneity of the individual consensus recognition sequences.

## Results

### Pax2 is required for the specification of otic precursors

**Fig. 1:**
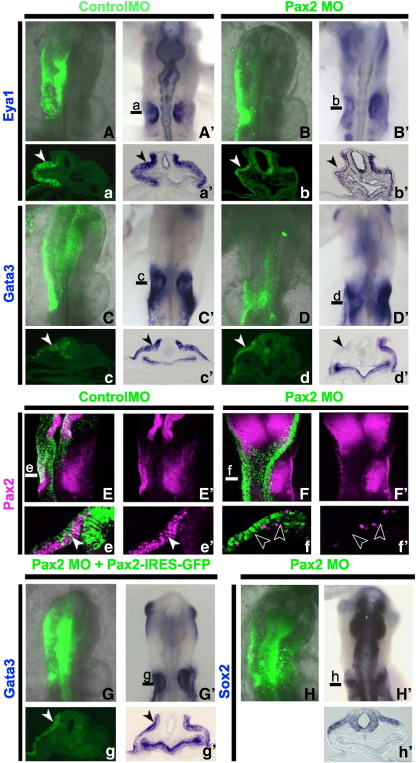
Pax2 is required for otic marker expression. Control (A, A′, C, C′, E, E′) or Pax2 MOs (B, B′, D, D′, F, F′, H H′) were electroporated into otic precursors at the 1–2 somite stage. At HH10–12, *Eya1* (A–B′, a–b′) and *Gata3* (C–D′, c–d′) expression is present in cells carrying control MOs (green in A, a, C, c; arrow heads), but absent in Pax2 MO electroporated cells (green in B, b, D, d; arrow heads). Note in d′: non-invaginated placode on targeted side. Pax2 protein expression is not affected by control MOs (E, E′, e, e′; arrow head), but absent in cells with Pax2 MOs (F, F′, f, f′; arrow head). Note the difference in cell shape of the control and Pax2 MO cells in e (white arrow head) and f (open arrow head). Loss of Pax2 does not affect *Sox2* expression in the otic placode (H, H′, h′). *Gata3* expression is rescued when otic cells are co-electroporated with splice blocking MOs and a Pax2 expression construct (G, G′, g, g′). Lines in A′–D′, E, F, G′ and H′ indicate the level of sections shown in a–g, a′–g′.

To establish whether Pax2 plays a role in the acquisition of otic fate in amniotes we designed a knock down approach in chick using two different morpholinos (MO). Otic identity was assessed by analysing the expression of the earliest pan-otic markers *Gata3*, *Eya1* and *Sox2*. Both MOs, one targeting the translation start site and the other targeting a splice junction, remove Pax2 protein effectively (Mende et al., 2008). When electroporated into the otic territory at HH6–8^−^ control MOs have no effect (Fig. 1A, A′, C, C′, E, E′), while both Pax2 MOs produce identical phenotypes: the expression of the otic placode markers *Gata3* (15/22), *Eya1* (6/10) and Pax2 (22/22) is abolished (Fig. 1B, B′, D, D′, F, F′), but *Sox2* expression is normal (n = 11; Fig. 1H, H′). To confirm that this effect is specific, we coexpressed Pax2 with the splice blocking MO and find that this rescues the loss of *Gata3* expression (5/5; Fig. 1G, G′). Thus, in chick Pax2 is necessary for the expression of some early otic-specific genes and thus may play a role in conferring otic identity to cranial ectoderm.

### Pax2 induces Gata3, but not other early otic markers

**Fig. 2:**
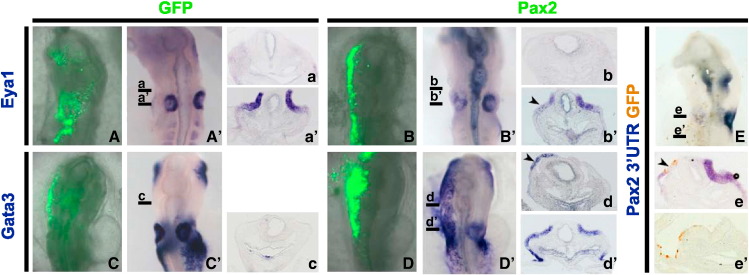
Pax2 is not sufficient to confer otic identify to ectodermal cells. Pax2-GFP (B, B′, D, D′, green; E, brown) or GFP (A, A′, C, C′, green) was misexpressed at the 0–1 somite stage. While *Gata3* expression is induced ectopically (D′, d, d′, arrow head), *Eya1* (B′, b′) and *Pax2* (E, e′) are not. Pax2 misexpression in the otic placode leads to loss of *Eya1* (B′, b; arrow head) and *Pax2* (E, e, arrow head), but expression of *Gata3* does not change (d). No effect is observed in control electroporated embryos (A′, a, a′, C′, c). Black lines in A′–E indicate the level of sections shown in a–e and a′–e′.

In the eye, the Pax family member Pax6 acts as a ‘master regulator’ inducing ectopic eyes when misexpressed (Gehring, 1996). To assess whether Pax2 has similar properties in the ear we misexpressed Pax2 at HH6/7. *Gata3* becomes dramatically upregulated in both the cranial (18/33; Fig. 2D, D′, d, d′) and trunk (not shown) ectoderm, while *Eya1* (n = 9; Fig. 2B, B′, b), *Sox2* (n = 8, not shown), *Sox3* (n = 7; not shown) and *Pax2* (n = 8; Fig. 2E, e′) are unaffected. We never observe ectopic placode-like structures or vesicles (see below) as seen with other transcription factors like Spalt4 (Barembaum and Bronner-Fraser, 2007). Surprisingly, Pax2 overexpression in the placode itself abolishes the expression of *Eya1* and *Pax2*, but not of *Gata3* (Figs. 2B′, b′, D′, d′, E, e). It is possible that the amount of Pax2 protein is critical for normal gene expression as suggested by the dose dependent function of Pax proteins in humans and mouse (for review see: Eccles et al., 2002). Alternatively, overexpression of Pax2 may sequester essential Pax2 co-factors and as a result downstream target gene expression is lost. Together, these observations suggest that Pax2 is not sufficient to impart otic character or placode morphology to non-otic ectoderm.

### Cell elongation and assembly of adherens junctions in the otic placode

**Fig. 3:**
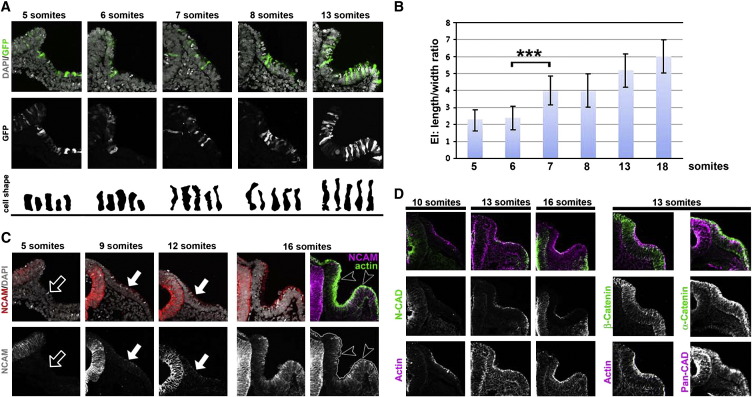
Cell shape changes during otic placode formation and assembly of the apical junctional complex. A. GPF was electroporated into otic precursors to visualise individual cells. Otic cells elongate dramatically after the 6 somite stage and continue to do so over the next hours. Bottom row: five representative cells from each stage to illustrate elongation. B. The elongation index (length/width ratio; EI) changes significantly between 6 and 7 somites (***p = 0.0007); mean values ± standard deviation are shown. C. N-CAM is absent in otic precursors at 5 somites (open arrow); it is first observed at the 9-somite stage (arrow) and intensifies thereafter. At 16 somites, double staining with phalloidin shows N-CAM (open arrow heads) localisation just basal to apical actin demarcated by the white line in bottom right panel. D. The components of adherens junctions cadherin, α-catenin and β-catenin assemble apically after the 10 somite stage.

Soon after otic-specific genes start to be expressed, otic precursor cells noticeably change their shape, as seen in many other morphogenetic events (Colas and Schoenwolf, 2001; Pilot and Lecuit, 2005; Schoenwolf and Franks, 1984; Wallingford, 2005). To visualise these changes in single cells, we electroporated GFP into the pre-otic domain to generate mosaic expression and analysed cell shapes by confocal microscopy. First, otic cells lengthen dramatically along their apical–basal axis, reflected by an increase in their elongation index (EI; length/width ratio) from 2.27 ± 0.67 (n = 8) and 2.36 ± 0.5 (n = 5) at the 5- and 6-somite stage respectively, to 4.03 ± 0.88 (p = 0.0007; n = 8) only 90 min later, at the 7 somite stage (Figs. 3A, B). Shortly thereafter (HH10), we observe apical accumulation of cortical actin (Fig. 3D; see also Sai and Ladher, 2008) and around the 13 somite stage, the key components of adherens junctions N-cadherin and α- and β-catenin (Knust and Bossinger, 2002; Niessen and Gottardi, 2008; Nishimura and Takeichi, 2009) assemble apically (Fig. 3D), while the cell adhesion molecule N-CAM accumulates just basal to the actin belt (Fig. 3C). Thus, cell elongation is followed rapidly by assembly of apical components necessary for apical constriction and subsequent invagination.

### Pax2, N-CAM and N-cadherin are essential for placode integrity

**Fig. 4:**
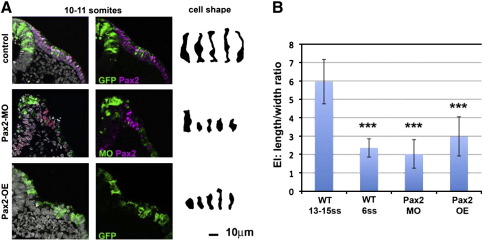
Pax2 controls cell shape in the otic placode. A. Otic precursors were electroporated with control MOs or GFP (top), Pax2 MOs (middle) or Pax2-GFP (bottom). While control cells have elongated at the 13–15 somite stage, Pax2 loss and Pax2 overexpression (Pax2 OE) result in loss of placode cell morphology. Right: five representative cells for each condition. B. Compared to control electroporated cells (WT 13–15ss) the EI is significantly reduced (***) in cells electroporated with Pax2 MOs or Pax2-GFP (Pax2 OE). For comparison measurements from 6 somite placodes are included (WT 6ss). Graph shows mean values ± standard deviation.

**Fig. 5:**
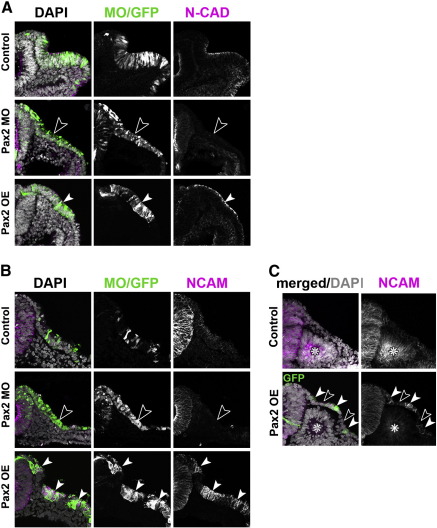
Pax2 is required for N-cadherin and N-CAM expression. A. Control electroporated otic placodes (top row) express N-cadherin (magenta) at the 15–16 somite stage. Loss of Pax2 (Pax2 MO, middle row) leads to loss of N-cadherin (open arrow heads, magenta), while N-cadherin is upregulated (white arrow heads) when Pax2 is misexpressed (Pax2 OE, bottom row). Note: N-cadherin is localised apically. B. Control electroporated otic placodes express N-CAM at the 12–13 somite stage (top row, magenta). Loss of Pax2 (Pax2 MO, middle row) leads to loss of N-CAM (open arrow heads, magenta). In contrast overexpression (Pax2 OE, bottom row) results in increased N-CAM (white arrow heads). Note: N-CAM is localised along the entire cell surface. C. N-CAM is not expressed in trunk ectoderm (control), however ectopic expression of Pax2 (Pax2 OE) in this tissue leads to upregulation of N-CAM in electroporated cells (green, white arrow heads), but not in non-electroporated neighbours (open arrow heads); *indicates somite.

In addition to changes in gene expression after gain or loss of Pax2 expression, we also observe changes in otic placode cell morphology (compare Figs. 1e and f; 2e). We therefore characterised shape changes in individual cells by electroporating Pax2 MOs into the pre-otic domain of HH6/7 embryos. Unlike their wild-type neighbours, targeted cells fail to maintain their columnar shape: their EI is significantly reduced when compared to cells carrying control MOs (Figs. 4A, B) and they resemble the ectoderm before placode formation (Pax2 MO: EI 2.03 ± 0.78, n = 9; control MO: EI 5.96 ± 1.21, n = 11; p = 0.0001; Fig. 4B). Since components of adherens junctions are required to maintain cell polarity and epithelial integrity (Nandadasa et al., 2009; Tinkle et al., 2008; Yang et al., 2009) we asked whether the lack of columnar morphology is accompanied by loss of N-cadherin and N-CAM. Indeed, both cell adhesion molecules are absent when Pax2 is knocked down (N-cadherin: n = 7; N-CAM: n = 7; Figs. 5A–C).

**Fig. 6:**
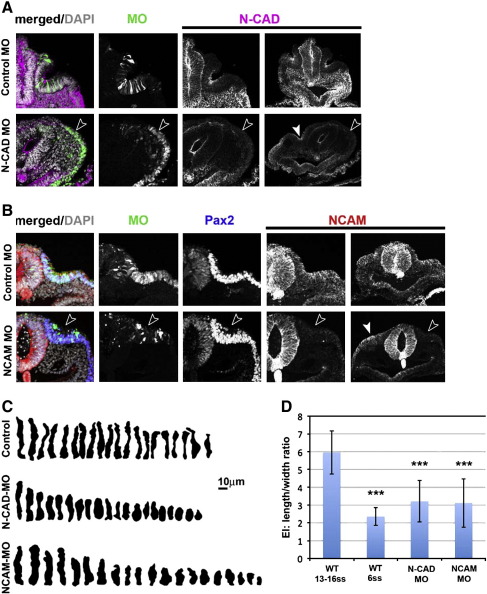
N-cadherin and N-CAM are required to maintain otic placode morphology. A. Loss of N-cadherin mimics the absence of Pax2. At the 16–18 somite stage, cells carrying control MOs show elongated shape (top row) and N-cadherin expression apically (magenta); panel on the right shows an overview of the same section with both placodes. In contrast, the placode does not thicken or invaginate in N-cadherin knock downs (bottom row, open arrow heads) and cells remain cuboidal. N-cadherin expression is lost (magenta) in the targeted side, but present on the contralateral side (panel on the right). B. Loss of N-CAM mimics the absence of Pax2. Control electroporated cells (top row) are elongated at the 13–15 somite stage and express N-CAM (red) and Pax2 (blue). In contrast, cells carrying N-CAM MOs (bottom row) are round (open arrow head), have lost N-CAM expression (red), but continue to express Pax2 (blue). Panels on the right show a low magnification to include the contralateral placode for comparison. C. Twenty representative cells from control, N-cadherin and N-CAM MO carrying cells show the difference in cell shape. D. The elongation index of cells expressing N-cadherin or N-CAM MOs is significantly reduced when compared to control electroporated cells (WT 13–16ss). For comparison the EI for placode cells from 6 somite embryos is included. Graph shows mean values ± standard deviation.

To assess whether loss of N-CAM or N-cadherin can phenocopy the loss of Pax2 and whether these molecules themselves are critical for placode integrity, we used MOs to knock down their expression in the ear. Otic cells carrying N-cadherin or N-CAM, but not control MOs, lose contact with their neighbours and fail to maintain an elongated, columnar shape (Fig. 6; control MO: n = 16; N-CAM MO: n = 15; N-cadherin MO: n = 10). At the 13–16 somite stage, their EI is significantly reduced compared to cells carrying control MOs (control: EI 5.7 ± 1.6; N-cadherin: EI 3.2 ± 1.2; N-CAM: EI 3.2 ± 1.3; Figs. 6C and D). Together, these results suggest that Pax2 controls integrity of the otic placode by regulating apical cell adhesion via N-CAM and N-cadherin and that both cell adhesion molecules are required independently to maintain cell elongation.

### Pax2 may control cell shape independent of otic fate

The above results show that Pax2 is required for expression of both otic markers and cell adhesion molecules, but is unable to induce ectopic otic fate. Is the acquisition of otic identity connected directly to the control of adhesive properties or cell shape? When Pax2 is expressed ectopically, both N-cadherin (n = 6) and N-CAM (n = 10) are strongly induced compared to control electroporated cells, even in trunk ectoderm (Figs. 5A, C). However, we do not observe cell elongation. This is probably due to aberrant subcellular localisation of N-CAM: while N-cadherin is restricted apically as in the normal otic placode, N-CAM fails to do so but is spread along the entire cell surface (Figs. 5B, C). When overexpressed in the otic placode itself, Pax2 also induces changes of cell morphology similar to those observed in the absence of Pax2 (Figs. 4A, B): their EI is reduced from 5.96 ± 1.21 in controls (see above) to 2.98 ± 1.06 (n = 15; p = 0.0001). Both N-cadherin and N-CAM (Figs. 5A, B) are upregulated, while otic markers are lost (see above Fig. 2). In addition, subcellular localisation of N-CAM is disturbed and placode organisation is disrupted (Fig. 5B). Thus, although Pax2 does not seem to be sufficient to initiate the otic programme in cranial ectoderm, it induces ectopic expression of N-cadherin and N-CAM in both locations. These findings suggest that Pax2 may control cell shape and otic identity through independent mechanisms.

### Sox2 is not sufficient to rescue otic cell shape in the absence of Pax2

**Fig. 7:**
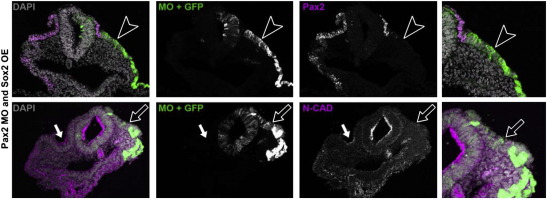
Sox2 is not sufficient to rescue cell shape or placode invagination in the absence of Pax2. Otic precursors were electroporated with Pax2 MOs and Sox2 at HH6/7. Cell shape and invagination of the otic placode remain disturbed: compare the non-electroporated control side (left) and the Pax2 MO/Sox2 expressing contralateral side (green, open arrow head or arrow). Pax2 expression (top row, magenta, open arrow head) is absent in electroporated cells; Sox2 does not rescue N-cadherin expression (bottom row, magenta, open arrow). Panels on the right show higher magnification of the targeted area.

An otic-specific enhancer for N-cadherin has recently been characterised, whose activity depends on SoxB1 group binding sites (Matsumata et al., 2005; see supplementary Fig. 1). In addition, we have identified two evolutionary conserved, putative Pax2 binding sites in this enhancer, one very close to a SoxB1 group binding site (Fig. S1). To assess if the control of N-cadherin by Pax2 is mediated by Sox proteins we electroporated otic precursors with Pax2 MOs together with full length Sox2. Sox2 is unable to rescue the Pax2 MO phenotype: the ectoderm remains cuboidal and N-cadherin is not expressed (Fig. 7). These findings show that Sox2 alone cannot restore N-cadherin expression and placode morphology in the absence of Pax2 function, suggesting that the factors may synergise to activate N-cadherin.

## Discussion

Pax2 is among the earliest genes to be expressed in the pre-otic field (Groves and Bronner-Fraser, 2000; Hans et al., 2004; Hidalgo-Sanchez et al., 2000; Streit, 2002; Torres et al., 1996). Here we show that in chick Pax2 plays a dual function as a key regulator of otic cell identity and shape. Pax2 function is required for the expression of otic transcription factors and for cell adhesion molecules, which in turn are necessary for epithelial integrity and subsequent placode invagination.

### Pax2 and otic precursor specification

Commitment of ectodermal cells to an otic fate is reflected by the sequential expression of transcription factors. Members of the Dlx and Foxi1 families initially demarcate the pre-otic field. In zebrafish they confer competence to respond to the otic inducing signal FGF (Hans et al., 2007; Nissen et al., 2003; Solomon and Fritz, 2002; Solomon et al., 2003, 2004). In response to FGFs, otic progenitors begin to express *Pax2* and *Pax8* (Hans et al., 2004; Martin and Groves, 2006; Wright and Mansour, 2003), which appear to cooperate in promoting otic development (Hans et al., 2004). In amniotes, however, a role for Pax proteins in otic specification has not yet been demonstrated. Pax2 mutant mice form an otic vesicle, but develop cochlear defects later (Torres et al., 1996; Burton et al., 2004), while otic development is merely delayed in Pax8 mutants (Christ et al., 2004); double mutants have not been examined. Since both Pax genes encode highly related transcription factors with common biochemical properties (Bouchard et al., 2000; Pfeffer et al., 1998), the lack of an early ear phenotype in either mutant is probably due to functional redundancy. Our finding that loss of chick Pax2 alone leads to the absence of early otic markers and of the placode itself seems to contradict the above results. However, it is possible that Pax2 is the only Pax gene expressed early during otic specification in birds.

The chromosomal region containing the Pax8 locus has undergone considerable chromosomal rearrangement during evolution (Fan et al., 2002a,b; Yunis and Prakash, 1982). In humans, the Pax8 locus is found on 2q13–2q14.1, a region that arose through fusion of two ancestral chromosomes (Yunis and Prakash, 1982) and analysis of the syntenic regions in mammals, amphibians and fish reveals frequent chromosomal rearrangements (AS, unpublished observations). While in amphibians, medaka and stickleback the Pax8-containing region clearly corresponds to that in mammals, the zebrafish Pax8 locus on chromosome 5 shows no synteny with this region. In birds and reptiles, however, the entire region is missing. It is therefore possible that the Pax8 locus was lost in Sauropsids, providing an explanation for why loss of Pax2 alone is sufficient to cause the loss of otic identity in chick. We therefore propose that in birds Pax2 is the key Pax protein controlling the specification of otic progenitor cells.

The Pax family member Pax6 plays a central role in eye formation and is able to induce ectopic eyes in many species across the animal kingdom (Gehring, 1996). Do other Pax proteins have similar functions as master regulators of sensory placode formation? *Pax3* is specifically expressed in the ophthalmic portion of the trigeminal placode (Stark et al., 1997). While it is required for the specification of trigeminal neurons, Pax3 is unable to induce them ectopically (Dude et al., 2009). Our results suggest that Pax2 alone may not be sufficient to impart otic character to non-otic ectoderm: *Gata3*, but none of the other otic markers tested, is upregulated in response to ectopic Pax2 expression. Thus, the ability to induce ectopic sensory structures appears to be unique to Pax6.

### The Sox, Pax and Gata cassette as coordinator of fate and morphogenesis?

Our results uncover a novel role for Pax2 in controlling placode morphology. When Pax2 expression levels are disturbed, otic cells fail to adopt columnar shape and instead remain cuboidal. The expression of two apically localised cell adhesion molecules, N-cadherin and N-CAM, is disrupted and as a consequence the placode epithelium loses integrity and fails to invaginate. Consistent with the idea that Pax2 regulates cell morphology and invagination, α-catenin, α-actinin and several microtubule associated proteins have been predicted as potential direct targets of Pax2 based on bioinformatic analysis (Ramialison et al., 2008). Within the Pax family, both Pax6 and Pax3 have been implicated in controlling cell adhesion, morphology and behaviour in the eye, neural crest cells and muscle (Buckingham and Relaix, 2007; Collinson et al., 2000; Edelman and Jones, 1995; Holst et al., 1997; Kallunki et al., 1995; Mayanil et al., 2000; Smith et al., 2009; Wiggan and Hamel, 2002). We therefore suggest that Pax proteins play a fundamental role in development by integrating cell fate allocation and morphogenetic events.

It is likely however that Pax proteins cooperate with other transcription factors to control placode morphogenesis. After *Pax2*, members of the SoxB1 family and *Gata3* become expressed in otic progenitors. Concomitantly, cells elongate to acquire columnar shape and then invaginate into an otic cup. In mouse, Sox9 and Gata3 are necessary for placode invagination (Barrionuevo et al., 2008; Lillevali et al., 2006) and we suggest that these factors, together with Pax2 and Sox2, control the expression of N-cadherin and N-CAM to maintain cell shape. The otic N-cadherin enhancer contains putative binding sites for all three factors (Fig. S1 and Matsumata et al., 2005) and they may therefore cooperate to initiate N-cadherin. Although the regulatory elements that control N-CAM expression in the ear have not been identified, other N-CAM enhancers contain Pax binding sites (Edelman and Jones, 1995; Holst et al., 1997).

Pax, Gata and SoxB1 group transcription factors are frequently coexpressed at sites where cell fate acquisition and morphogenesis are tightly controlled (Barrionuevo et al., 2008; Grote et al., 2006; Lillevali et al., 2006; Matsumata et al., 2005; Rajakumar and Chamberlin, 2007; Smith et al., 2009). SoxB1 and Pax proteins often synergise to control gene expression. In the lens, Sox2 and Pax6 control *δ-crystallin* and *N-cadherin* (Matsumata et al., 2005; Smith et al., 2009) and are coexpressed with *Gata3* (see Figs. 1 and 2; Sheng and Stern, 1999). Likewise, they control the activity of the diencephalic enhancer (N3) of Sox2 (Inoue et al., 2007), while Sox9 and -10 synergise with Pax3 to activate neural crest and glia expression of Sox10 (Werner et al., 2007). Although the role of Gata proteins in this context is less well established, these factors are essential for endoderm invagination in C. elegans (Sawyer et al., 2010). Thus, Pax, SoxB1 and Gata factors may emerge as key coordinators of cell behaviour and fate.

### Cell adhesion molecules in otic cell morphology and invagination

Our studies reveal an essential role for two cell adhesion molecules, N-CAM and N-cadherin, in the maintenance of epithelial integrity and invagination of the otic placode. While N-CAM plays important roles in the developing and adult nervous system being involved e.g. in neurite outgrowth, synaptic plasticity and regeneration (Ditlevsen et al., 2008; Edelman, 1985; Maness and Schachner, 2007), little is known about its potential role in epithelial morphogenesis. N-CAM is best known for its function as homophilic cell adhesion molecule in neuronal cells, but recent evidence suggests that it also acts as a multifunctional regulator of cell behaviour (Ditlevsen et al., 2008; Hansen et al., 2008 and references therein). It regulates cytoskeletal dynamics by associating with proteins like spectrin, α- and β tubulin and α-actinin and by coupling membrane associated complexes to the cytoskeleton. It is tempting to speculate that interactions similar to those that regulate neurite outgrowth also modulate epithelial cell behaviour.

Cadherin-based adherens junctions are crucial for remodelling and folding of epithelial sheets, for maintaining cell polarity and for tissue integrity (D'Souza-Schorey, 2005; Gumbiner, 2005; Nishimura and Takeichi, 2009). Transmembrane cadherins attach to cortical actin through α-, β- and γ-catenin (Hirano et al., 1987; Matsuzaki et al., 1990; Nagafuchi and Takeichi, 1988; Ozawa et al., 1989), but are also actively involved in the assembly of cortical F-actin (Nandadasa et al., 2009). Cadherins therefore drive morphogenetic tissue movements such as apical constriction in the neural tube. Our results support the idea that N-cadherin, downstream of Pax2, plays a similar role in placode invagination. During neurite outgrowth, N-CAM and N-cadherin interact with common and distinct intracellular partners and both modulate FGF-receptor signalling (for review see: Hansen et al., 2008). In the otic placode, FGF signalling is critical for apical actin accumulation (Sai and Ladher, 2008), raising the possibility that N-CAM and N-cadherin not only mediate cell–cell adhesion, but may also influence the signalling pathways involved in invagination.

In conclusion, our studies suggest that during development transcriptional regulators like Pax proteins play a critical role not only in assigning cell fate, but also in controlling morphogenetic events. Pax proteins together with Sox and Gata factors may therefore provide the missing link between signalling pathways that induce cell identity and shaping of complex organs.

## Appendix A Supplementary data

Supplementary Figure 1 ClustalW alignment of human, mouse and chicken *N-Cadherin* En2-DP enhancer sequences. Shaded bases are conserved across all three species, as reflected in the consensus enhancer sequence. The consensus is annotated with putative conserved transcription factor binding sites: those above the consensus are encoded on the sense strand, whereas those below are encoded on the anti-sense strand. Shaded bases within the annotated binding sites are conserved across all three species. The lightly-shaded box indicates the position of a putative paired Pax2/SoxB1 recognition site, reminiscent of the chicken *delta-Crystallin* (DC5) and *Sox2* (N3) enhancers, which are synergistically activated by Pax6 and Sox2 in the lens placode.
