# Supplementary material for: Pax2 coordinates epithelial morphogenesis and cell fate in the inner ear
Source: Dev Biol. 2010 Sep 15;345(2-3):180–90. doi: 10.1016/j.ydbio.2010.07.007 (PMC2946559; doi:10.1016/j.ydbio.2010.07.007)
Supplement: Supplementary Figure 1 — ClustalW alignment of human, mouse and chicken N-Cadherin En2-DP enhancer sequences. Shaded bases are conserved across all three species, as reflected in the consensus enhancer sequence. The consensus is annotated with putative conserved transcription factor binding sites: those above the consensus are encoded on the sense strand, whereas those below are encoded on the anti-sense strand. Shaded bases within the annotated binding sites are conserved across all three species. The lightly-shaded box indicates the position of a putative paired Pax2/SoxB1 recognition site, reminiscent of the chicken delta-Crystallin (DC5) and Sox2 (N3) enhancers, which are synergistically activated by Pax6 and Sox2 in the lens placode. [file mmc1.pdf]

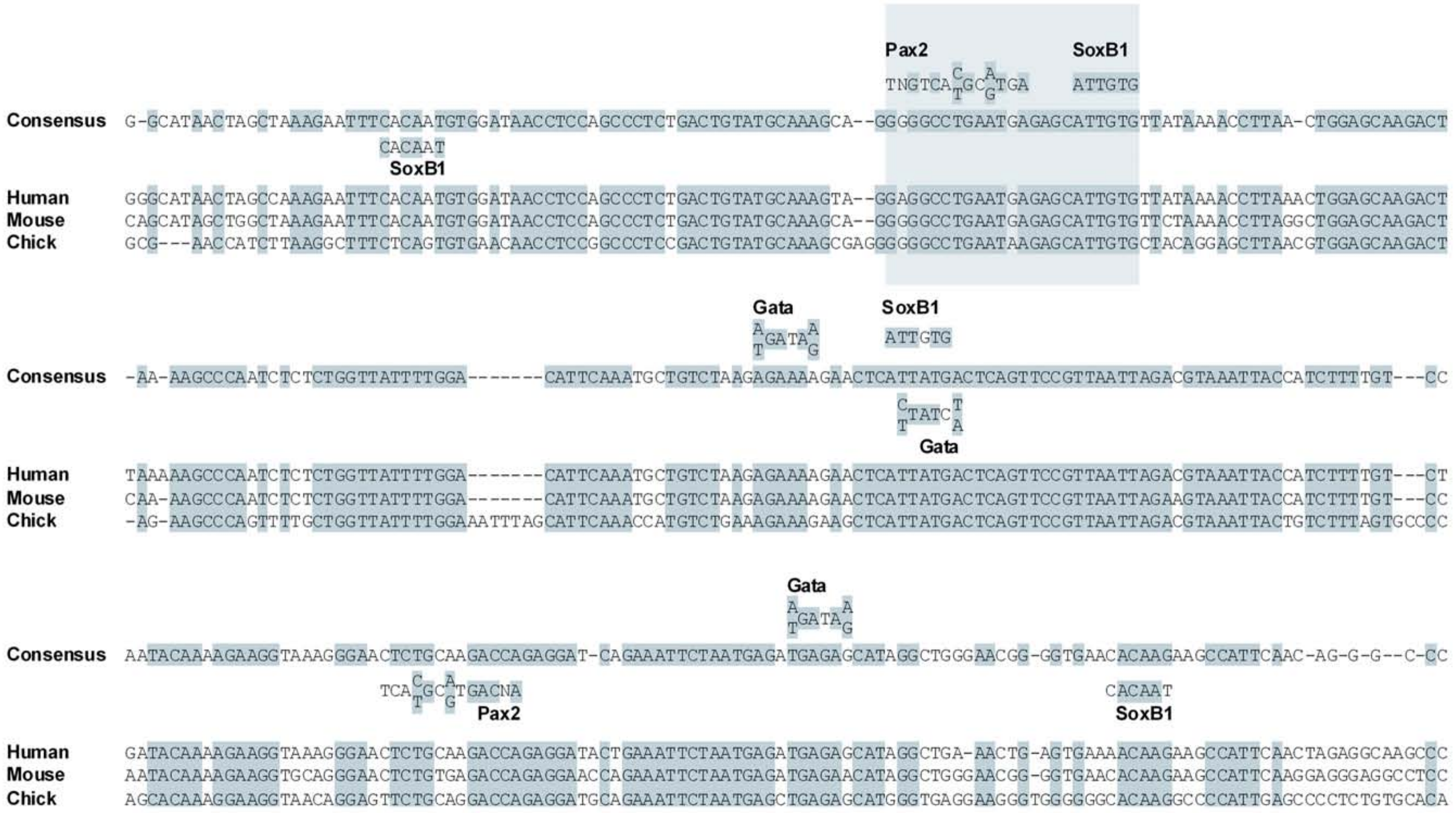
Pax2
TNGTCA^C_TGC^A_GTGA
SoxB1
ATTGTG
Consensus G-GCATAACTAGCTAAAGAATTTCACAATGTGGATAACCTCCAGCCCTCTGACTGTATGCAAAGCA--GGGGGCCTGAATGAGAGCATTGTGTTATAAAACCTTAA-CTGGAGCAAGACT
CACAAT
SoxB1
Human GGGCATAACTAGCCAAAGAATTTCACAATGTGGATAACCTCCAGCCCTCTGACTGTATGCAAAGTA--GGAGGCCTGAATGAGAGCATTGTGTTATAAAACCTTAAACTGGAGCAAGACT
Mouse CAGCATAGCTGGCTAAAGAATTTCACAATGTGGATAACCTCCAGCCCTCTGACTGTATGCAAAGCA--GGGGGCCTGAATGAGAGCATTGTGTTCTAAAACCTTAGGCTGGAGCAAGACT
Chick GCG---AACCATCTTAAGGCTTTCTCAGTGTGAACAACCTCCGGCCCTCCGACTGTATGCAAAGCGAGGGGGGCCTGAATAAGAGCATTGTGCTACAGGAGCTTAACGTGGAGCAAGACT
Gata
^A_TGATA^A_G
SoxB1
ATTGTG
Consensus -AA-AAGCCCAATCTCTCTGGTTATTTGGA-------CATTCAAATGCTGTCTAAGAGAAAAGAACTCATTATGACTCAGTTCCGTTAATTAGACGTAAATTACCATCTTTTGT---CC
^C_TTATC^T_A
Gata
Human TAAAAAGCCCAATCTCTCTGGTTATTTGGA-------CATTCAAATGCTGTCTAAGAGAAAAGAACTCATTATGACTCAGTTCCGTTAATTAGACGTAAATTACCATCTTTTGT---CT
Mouse CAA-AAGCCCAATCTCTCTGGTTATTTGGA-------CATTCAAATGCTGTCTAAGAGAAAAGAACTCATTATGACTCAGTTCCGTTAATTAGAAGTAAATTACCATCTTTTGT---CC
Chick -AG-AAGCCCAGTTTTGCTGGTTATTTGGAAATTTAGCATTCAAACCATGTCTGAAAGAAAGAAGCTCATTATGACTCAGTTCCGTTAATTAGACGTAAATTACTGTCTTTAGTGCCCC
Gata
^A_TGATA^A_G
Consensus AATACAAAAGAAGGTAAAGGGAACTCTGCAAGACCAGAGGAT-CAGAAATTCTAATGAGATGAGAGCATAGGCTGGGAACGG-GGTGAACACAAGAAGCCATTCAAC-AG-G-G--C-CC
TCA^C_TGC^A_GTGACNA
Pax2
CACAAT
SoxB1
Human GATACAAAAGAAGGTAAAGGGAACTCTGCAAGACCAGAGGATACTGAAATTCTAATGAGATGAGAGCATAGGCTGA-AACTG-AGTGAAAACAAGAAGCCATTCAACTAGAGGCAAGCCC
Mouse AATACAAAAGAAGGTGCAGGGAACTCTGTGAGACCAGAGGAACCAGAAATTCTAATGAGATGAGAACATAGGCTGGGAACGG-GGTGAACACAAGAAGCCATTCAAGGAGGGAGGCCTCC
Chick AGCACAAAGGAAGGTAACAGGAGTTCTGCAGGACCAGAGGATGCAGAAATTCTAATGAGCTGAGAGCATGGGTGAGGAAGGGTGGGGGCACAAGGCCCCATTGAGCCCCTCTGTGCACA

Supplementary Figure 1. ClustalW alignment of human, mouse and chicken *N-Cadherin* En2-DP enhancer sequences.

Shaded bases are conserved across all three species, as reflected in the consensus enhancer sequence. The consensus is annotated with putative conserved transcription factor binding sites: those above the consensus are encoded on the sense strand, whereas those below are encoded on the anti-sense strand. Shaded bases within the annotated binding sites are conserved across all three species. The lightly-shaded box indicates the position of a putative paired Pax2/SoxB1 recognition site, reminiscent of the chicken *delta-Crystallin* (DC5) and *Sox2* (N3) enhancers, which are synergistically activated by Pax6 and Sox2 in the lens placode.
